# Neonatal Seizures Revisited

**DOI:** 10.3390/children8020155

**Published:** 2021-02-18

**Authors:** Konrad Kaminiów, Sylwia Kozak, Justyna Paprocka

**Affiliations:** 1 Students’ Scientific Society, Department of Pediatric Neurology, Faculty of Medical Sciences in Katowice, Medical University of Silesia, 40-752 Katowice, Poland; kaminiow.k@gmail.com (K.K.); sylwiakozak@icloud.com (S.K.); 2 Department of Pediatric Neurology, Faculty of Medical Sciences in Katowice, Medical University of Silesia, 40-752 Katowice, Poland

**Keywords:** neonatal seizures, pathophysiology, genetics, inborn errors of metabolism

## Abstract

Seizures are the most common neurological disorder in newborns and are most prevalent in the neonatal period. They are mostly caused by severe disorders of the central nervous system (CNS). However, they can also be a sign of the immaturity of the infant’s brain, which is characterized by the presence of specific factors that increase excitation and reduce inhibition. The most common disorders which result in acute brain damage and can manifest as seizures in neonates include hypoxic-ischemic encephalopathy (HIE), ischemic stroke, intracranial hemorrhage, infections of the CNS as well as electrolyte and biochemical disturbances. The therapeutic management of neonates and the prognosis are different depending on the etiology of the disorders that cause seizures which can lead to death or disability. Therefore, establishing a prompt diagnosis and implementing appropriate treatment are significant, as they can limit adverse long-term effects and improve outcomes. In this review paper, we present the latest reports on the etiology, pathomechanism, clinical symptoms and guidelines for the management of neonates with acute symptomatic seizures.

## 1. Introduction

Acute symptomatic seizures are the most common neurological emergency in newborns and are most prevalent in the neonatal period. They are early signs of brain damage and can severely disturb the development of the infant’s immature brain. Seizures affect 1–3 neonates per 1000 live births [1,2]. The prevalence is much higher in premature neonates (10–130/1000 live births) [3,4,5]. Neonatal seizures are defined as a paroxysmal electroclinical phenomenon characterized by the transient occurrence of signs and symptoms due to an abnormal excessive or synchronous neuronal activity in the brain [6]. By the traditional definition, neonatal seizures occur in the first 28 days after birth of a term neonate or before 44 weeks of gestational age in a preterm neonate [6]. The etiology depends on whether the patient is a term or premature neonate. In the former group, the most common cause is hypoxic-ischemic encephalopathy (HIE), and in the latter group, intracranial hemorrhage (ICH) is most prevalent [4]. Despite the fact that neonatal seizures are a rare neurological disorder, they can lead to serious health consequences. They have an extremely detrimental effect on the developing brain and can result in cognitive disorders, developmental delay, epilepsy or cerebral palsy [6,7,8,9]. Therefore, it is crucial to start the diagnostic process immediately after the suspicion of seizures and initiate effective treatment. However, the diagnosis poses a challenge for clinicians, as most seizures can be clinically subtle and difficult to identify [10]. Moreover, while neonatal seizures can occur with or without clinical manifestations, the majority of neonatal seizures (50–80%) are electrographic only without clinical manifestations [11]. As a result, conventional EEG monitoring is the gold standard in the diagnosis of neonatal seizures [11,12,13]. Considering the number of many serious consequences, treatment of neonatal seizures is necessary. Phenobarbital, phenytoin, or levetiracetam are the antiepileptic drugs commonly used for the treatment of neonatal seizures. However, caution should be exercised with the use of these agents, since each of them has contraindications and may increase the risk of adverse effects. 

The paper presents a summary of the results of the latest research on neonatal seizures. The collected data were divided into sections, representing various aspects related to the disorder, i.e., pathophysiology, etiology, spectrum of symptoms, classification, diagnosis, treatment and outcomes.

## 2. Materials and Methods

The search strategy consisted of controlled vocabulary and keywords. The following databases were searched: PubMed, Medline and Google Scholar. The main search concept was to combine “neonatal seizures” with related terms, such as “pathophysiology”, “etiology”, “genetic(s)”, “symptoms”, “diagnosis”, “treatment” and “outcomes”. Filters which were applied to limit the retrieval included the language and the date of publication. For this reason, only English language papers published within the previous 10 years were considered for this review. Exclusion criteria included articles not containing the related terms, articles written in a language other than English or articles published more than 10 years ago. Each database was searched individually, and search terms were applied line by line and were replicated in every source. The entire process of searching relevant papers lasted from April 2020 to October 2020, with numerous subsequent updates on the latest scientific reports. Titles, abstracts and full-text articles were screened against the inclusion criteria by two reviewers. Next, manual search and reference and citation tracking were undertaken by two reviewers (KK and SK) who established the final selection of papers. Any disagreement was resolved by discussion. In the case of no agreement, a third independent reviewer made the final decision. Throughout the search process, 57 articles were found and 40 of them were included in the final analysis. The articles included in the review were grouped into the neonatal seizure-related thematic categories for better organization, e.g., “etiology”, ”diagnosis” or “outcomes”. 

## 3. Results

### 3.1. Pathophysiology

In the mature brain, gamma aminobutyric acid (GABA), which is the main inhibitory messenger, induces the influx of chloride ions and hyperpolarization of a neuronal membrane after binding of the GABA receptor agonist, which makes the neuron unable to conduct the impulse. This is due to high expression of potassium-chloride cotransporter 2 (KCC2) and low expression of sodium-potassium-chloride cotransporter 1 (NKCC1). Preclinical models showed that in the immature brain of newborns, the proportion of cotransporter expression was different [14]. Due to higher expression of NKCC1 compared to KCC2 in neonates, when GABA binds to the GABA_A_ receptor, the outflow of chloride ions and depolarization of the neuronal cell membrane increases, which implies higher sensitivity of neurons to stimulation and generation of action potential [15]. These structural changes affect the functioning of the immature brain of the newborn and imply greater susceptibility to seizures by increasing the mechanisms facilitating stimulation and decreasing their inhibition [15]. When GABA binds to the GABA_A_ receptor in the neonate’s brain where NKCC1 is predominant, depolarization caused by chloride ion efflux also triggers internal calcium currents and removes the voltage-dependent magnesium block of N-methyl D-aspartate (NMDA) receptors [16,17]. This promotes the entry of calcium ions and the activation of second messengers, which increases brain excitability and the risk of seizures [16,17]. Additionally, perinatal injuries (e.g., ischemia) increase NKCC1 expression, while reduction in KCC2 and HIE can cause an isolated increase in NKCC1 [18]. 

The process of a dynamic increase in the number and density of synapses and dendrites (synaptogenesis), which occurs in the neonatal period, is also important in the pathophysiology of seizures. Then, physiologically enhanced excitation in the neonatal period is necessary for the proper development of newly formed neurons (their differentiation, migration and formation of synapses). However, it also makes the neonatal brain more susceptible to seizures [6,19].

### 3.2. Etiology

Neonatal seizures are a nonspecific heterogeneous symptom of brain injury that has many different causes. Etiology of seizures depends on the gestational age of the newborn. Among term neonates and late premature neonates (>33 weeks’ gestational age; wGA), the most common causes of brain damage manifested by seizures include HIE, ischemic stroke, ICH, transient metabolic and electrolyte disturbances, systemic or central nervous system (CNS) infections [2,20,21]. The causes also include congenital malformations of the CNS and genetic epilepsy syndromes (e.g., benign familial neonatal seizures or inborn errors of metabolism) [2,20,21]. In the group of premature and extremely premature neonates (<28 wGA), intracranial hemorrhage and its complications are the dominant cause of brain damage that results in seizures [4]. There is much evidence for a strong correlation between low gestational age, low birth weight and the occurrence of seizures in neonates [22]. The relationship between prematurity, brain damage and the occurrence of neonatal seizures was also demonstrated [22,23]. 

HIE is the most common cause of acute seizures and accounts for approximately 40% of all neonatal seizures [2,24]. Among full-term newborns with HIE, hypothermia [17,25] is the standard method of prophylaxis against seizures [17,25]. However, it was proven that despite the use of this method, seizures were still reported in some neonates [26,27]. Ischemic stroke is another leading cause, responsible for approximately 18% of all neonatal seizures [2]. Most ischemic strokes involve an area vascularized by the middle cerebral artery and are caused by embolism from the placenta, the umbilical cord or the heart [17]. There are many risk factors for developing ischemic stroke in neonates. Both neonatal factors (e.g., congenital heart defects, coagulation disorders, infections) and maternal factors (e.g., chorioamnionitis, premature rupture of membranes, oligohydramnios, diabetes) are involved [17,28,29,30]. Among many causes, ICH accounts for more than 10% of seizures [2] and is the dominant cause of prematurity [4]. The occurrence of seizures is caused by malformations of cerebral vessels, coagulation disorders or traumatic labor. Also, transient electrolyte and biochemical disturbances in sodium, calcium or glucose levels can lead to neonatal seizures which usually resolve after restoring the water-electrolyte balance. However, it is important to diagnose the primary cause of the disorders in order to manage patients and minimize the risk of seizure recurrence [31]. In some cases, seizures are also caused by systemic or CNS infections. Congenital viral infections (e.g., herpes simplex virus, cytomegalovirus or rotavirus) and bacterial infections (mainly caused by group B streptococci or *Escherichia coli*) can also be involved [17]. Due to the fact that meningitis is a common cause of seizures, lumbar puncture is recommended in neonates with seizures. Empirical antibiotic therapy or antiviral medications should be started immediately if the condition of the neonate is not stable [17]. Congenital malformations of the CNS (e.g., tuberous sclerosis or focal cortical dysplasia), inborn errors of metabolism, which are a heterogeneous group of diseases, or genetically determined epilepsy syndromes are characterized by a genetic component [20,32]. Such cases are rare, but due to the risk of irreversible neurological damage, the diagnostic process should be started immediately, and appropriate treatment should be initiated [20]. Considering the inheritance of certain disorders, such as benign familial neonatal seizures, parents of neonates should be provided with genetic counselling. 

Although there are many causes of neonatal seizures, clinicians should be vigilant when establishing the diagnosis, bearing in mind the fact that about 50% of neonates have more than one cause of seizures [33]. Due to various etiology, there are differences in the onset of neonatal seizures between full-term and preterm neonates, in whom seizures usually occur at a later stage [4,23]. Of note, differences are also found even in a group of premature neonates. One study showed that the mean time of seizure onset was 10.8 days in patients born <29 wGA compared to 5.2 days in patients born >29 wGA [23]. The list of the most common causes of neonatal seizures with the approximate period of seizure onset is given in Table 1. Data presented in Table 1 are based on three clinical trials conducted at different time points. The first study on a group of 221 newborns was carried out between 2002 and 2009. Another study was conducted between 2009 and 2013 and evaluated a group of 378 newborns. The third research was conducted between 2013 and 2015 was related to a group of 426 neonates. Unfortunately, there are no more recent studies available than the one conducted in 2013–2015.

**Figure 1:**
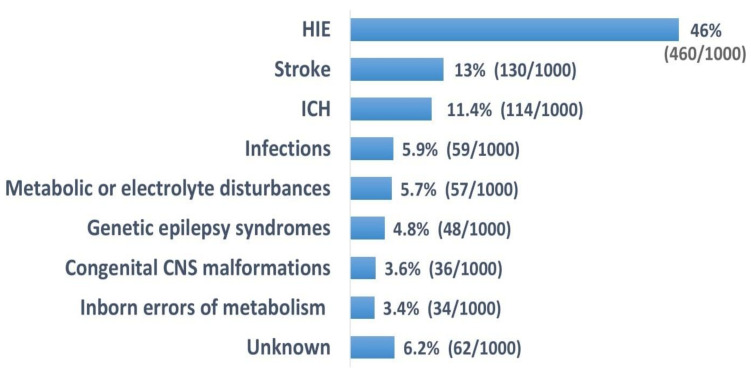
Most common causes of neonatal seizures [2,24,31].

The percentage of each cause of seizures varies depending on the study. For this reason, we decided to average the data from these studies and present a schematic diagram that allowed us to visualize the most common causes of neonatal seizures more easily (Figure 1).

### 3.3. Genetics of Neontal Seizures

The knowledge of seizures and their etiology has been growing for many years. It is already known that some causes of neonatal seizures are characterized by a genetic basis [35]. It is estimated that it concerns about 15% of neonates who present with distinct neonatal epilepsy syndromes related to brain malformations or genetic etiologies [1,36]. These disorders can affect the onset of seizures in neonates in many different respects. To better understand and systematize knowledge, these disorders can be divided into categories depending on the type of disorder. Among the categories of genetic causes, the following are distinguished: structural brain malformations, inborn errors of metabolism (i.e., due to hypoglycemia or the accumulation of toxic substances and metabolites), syndromic and nonsyndromic, single gene (including channelopathies and genes involved in the metabolism of neurotransmitters) [37,38].

#### 3.3.1. Structural Brain Malformation

Developmental defects of the cerebral cortex account for approximately 4% of all neonatal seizures [2,24,34] and are mostly related to corpus callosum agenesis (ACC), polymicrogyria, lissencephaly, schizencephaly, hemimegalencephaly, focal cortical dysplasia, tuberous sclerosis, holoprosencephaly and subcortical band heterotopia [20,31,37]. These disturbances may be the result of the disruption of embryogenesis, i.e., abnormal cell proliferation and migration, or a cortical organization, and are usually caused by pathogenic variants of genes that are precisely defined and easy to identify (e.g., *PAFAH1B1, TSC1* and *TSC2*, *DCX*, *ARX*, *DEPDC5*) [20,31,37]. These may be isolated defects or components of genetic syndromes. Therefore, it is important to search for other extra cerebral disorders in different organs [31]. The onset of neonatal seizures caused by structural brain malformation can vary significantly [31,36]. Abnormal brain structures can be seen even in utero on MRI. However, the diagnosis should be confirmed with postnatal brain MRI [31]. Rapid diagnosis is crucial because in many cases, seizures can be resistant to treatment and the defects are associated with poor neurodevelopment, and the delay ineffective treatment may significantly worsen the outcomes [20,31]. Intrauterine disorders (e.g., an infection or hypoxia) that affect hypoxia-sensitive cells may imitate anomalies due to genetic defects [20,37]. Therefore, an accurate assessment of the etiology is necessary. It has a significant impact on genetic counseling for parents related to subsequent pregnancies. 

#### 3.3.2. Inborn Errors of Metabolism

Inborn errors of metabolism are most often suspected on the basis of clinical manifestations (seizures, poor feeding and lethargy) and biochemical test results (often including wide anion gap, acidosis, hypoglycemia, ketonuria or hyperammonemia) [20,39]. The assessment of the disorder by genetic testing is a major challenge, as these defects should be immediately identified to avoid metabolic decompensation [1,40,41], particularly due to the fact that typical antiseizure medications (ASMs) may be ineffective in this case [31].

Pyridoxine-dependent epilepsy (mutations in *ALDH7A1* and *PROSC* genes) is one of the most common inborn errors of metabolism that results in neonatal seizures. It is an autosomal recessive disorder that manifests as a result of deficiency of α-aminoadipic semialdehyde dehydrogenase in the lysine degradation pathway [31,37,42]. It leads to a buildup of α-aminoadipic semialdehyde, piperideine-6-carboxylate and pipecolic acid [37,42]. When this disorder is suspected, the pyridoxine trial can be used. It involves the intravenous administration of 100 mg vitamin B6 to a newborn [31,37]. This trial should be performed under EEG control. Monitoring should be performed before, during and after the administration of the vitamin. Oxygen support therapy should be provided in case of apnea, which can be an adverse reaction [31,42,43]. Oral administration of vitamin B6 liquid formulation (2 × 50 mg/kg, 2–3 times daily for two or three days) is an alternative to intravenous drug administration [44,45]. After administration of vitamin B6, seizures should disappear within a few minutes. Some patients require a repeat dose, after which clinical and electroencephalographic symptoms subside. It is recommended to administer pyridoxine until pyridoxine-dependent epilepsy (PDE) is ruled out by biochemical and/or molecular testing [44,45]. Seizures can be well-controlled with pyridoxine supplementation. However, the developmental delay can still persist [37]. In another disorder known as pyridoxine-5′-phosphate oxidase deficiency, which is associated with a mutation in the *PNPO* gene, neonates may also respond well to vitamin B6, although they still have similar clinical manifestations [31,42]. Other relatively frequent disorders include biotinidase deficiency (mutation in the *BTD* gene, autosomal recessive inheritance), nonketotic hyperglycinemia (mutation in the *GLDC*, *AMT*, *GCSH* genes; autosomal recessive mutations), molybdenum cofactor and sulfite oxidase deficiency (mutations in the *MOCS1* and *MOCS2* genes, as well as in the *GPHN* gene responsible for cofactor deficiency or in the *SUOX* gene responsible for oxidase deficiency) [31,37,43,46]. In the case of molybdenum cofactor deficiency, neuroimaging studies can be of great significance. Initially, only edema is seen on MRI, but later, cystic leukoencephalopathy with cortical atrophy can be found [1,47,48]. Other metabolic causes may be suspected if specific clinical features are present and raise suspicion. Determination of biomarkers in blood, urine or cerebrospinal fluid, whose configurations are characteristic of a given defect, can significantly simplify and shorten the diagnostic process. The profile of biomarkers determined in common metabolic disorders is presented in Table 2. Metabolic screening tests are performed in neonates within the first hours after birth in many countries. They can exclude or confirm a metabolic defect even if it is still asymptomatic.

#### 3.3.3. Syndromic Disorders

Seizures in neonates can often manifest as part of the genetic syndrome, which commonly affects many systems, and the characteristic dysmorphic features are evident. Such syndromes can be divided according to the type of disease. The following can be distinguished among chromosomal disorders associated with neonatal seizures: Down syndrome, Patau syndrome, Edwards syndrome, Wolf–Hirschorn syndrome and the 22q11.2 deletion syndrome [37]. It was also found that neurocutaneous syndromes may initially manifest as neonatal seizures [37]. Table 3 shows the most common genetic syndromes in which seizures occur as one of the symptoms.

#### 3.3.4. Nonsyndromic Disorders

Neonatal seizures can also occur as main symptoms resulting from mutations in one gene, which is mainly related to genes involved in the regulation of ion channels, synaptic function and intercellular signaling [31,37]. Chanellopathies may usually result from disturbances in the *KCNT1, KCNQ2, CACNA1A* or *SCN2A* genes, which are responsible for the regulation of calcium, sodium or potassium channels [31,37,49,50]. Of note, some forms of neonatal seizures have a familial occurrence [20,32,51,52]. This group includes benign familial neonatal seizures, which are a rare, autosomal dominant inherited form [20,32,51,52] characterized by seizures occurring within the first week of life with a strong family history of neonatal seizures. This type of seizures is caused by mutations in the *KCNQ2* and *KCNQ3* genes, which are responsible for voltage-gated potassium channels and one chromosomal inversion [20,32,51,52]. Changes in the functioning of synapses or communication between cells are often caused by pathogenic variants in the *STXBP1, TBC1D24, SIK1, CDKL5* and *BRAT1* genes [31,49,50]. Some of the clinical syndromes caused by these mutations are mild and self-limiting, whereas others are severe [31]. These syndromes include self-limited neonatal seizures, self-limited neonatal familial epilepsy, early infantile epileptic encephalopathy (EIEE), early myoclonic encephalopathy (EME) and epilepsy of infancy with migrating focal seizures (EIMFS) [31]. 

Self-limited neonatal seizures are also known as "fifth day fits" due to the time of their occurrence (usually between the 4th and 6th days of life) [31,53]. Seizures can sometimes cause apnea and lead to status epilepticus [31]. Remission is reported within two days [31,53]. In turn, self-limited familial neonatal epilepsy usually occurs earlier than the above syndrome and remission is expected by six months of age [31,53]. Due to autosomal dominant inheritance, a positive family history of neonatal seizures provides valuable information and leads to suspicion of this syndrome [31,53,54]. Early infantile epileptic encephalopathy (EIEE), also known as Ohtahara syndrome, occurs in the first three months of life, often in the first two weeks [31]. It is associated with structural brain malformations and metabolic disorders [31]. Many genes whose mutations lead to EIEE have been recognized (*ARX, CDKL5, SLC25A22, STXBP1, KCNQ2, SPTAN1* and *SCN2A)* [31,35]. Patients with this syndrome may progress from EIEE to West syndrome, and later to the Lennox–Gastaut syndrome [31,55]. EIEE is similar to early myoclonic encephalopathy (EME). However, they may differ in terms of seizure semiology [31]. EME is often associated with metabolic disorders and mutations in the *STXBP1, TBC1D24* and *GABRA1* genes [31,43,53]. EIMFS is caused by mutations in the *KCNT1, SCN2A, SCN1A, SLC25A22, PLCB1, TBC1D24* and *QARS* genes [31,56]. The characteristics of the types of neonatal epilepsy are given in Table 4.

Despite the development of molecular diagnostics, the correlation between genotype and phenotype still poses some difficulty. As a result, the same genetic syndrome can be caused by variants in different genes, and abnormal gene variants can lead to different clinical phenotypes in different neonates [57].

### 3.4. Symptoms and Semiology Classification

Seizures are the most common paroxysmal, stereotypical and repetitive neurological events. Therefore, any abnormal behavior in neonates should be considered a potential seizure, requiring confirmation by electroencephalography (e.g., video-EEG). In addition to typical symptoms such as tonic, clonic and myoclonic seizures, other subtle manifestations of clinical seizures are reported (e.g., horizontal nystagmus, eyelid blinking, eyelid flutter, staring, chewing, sucking and munching) [58]. Sometimes a newborn can also present with movements imitating swimming, pedaling or boxing. Apnea and changes in blood pressure are also reported [58]. Clinically silent seizures are also very common. It is estimated that even 80–90% of neonates with electroencephalographically confirmed seizures may not have clinical symptoms [10]. They occur until ictal discharges affect the motor cortex [17,59].

A wide range of etiologies of neonatal seizures indicates high variability in seizure types and poses a diagnostic challenge for clinicians. In 2017, the International League Against Epilepsy (ILAE) introduced a revised classification of seizure types [60]. This classification is not only related to neonatal seizures, but includes seizures that affect patients of all age groups. Of note, a separate Task Force for neonatal seizures developed specific guidelines for neonates [11]. Seizures were defined as the transient occurrence of signs and/or symptoms caused by abnormal excessive or synchronous neuronal activity in the brain [11,60]. The ILAE Task Force classified neonatal seizures based on clinical symptoms and EEG, depending on the predominant seizure type: clinical (only abnormal, stereotypical behavior, without EEG changes), electroclinical (pathological signs including changes in electrographic activity) and only electrographic seizures (no clinical signs) [6,11]. Subclinical seizures mostly occur in neonates with encephalopathy and in critical medical condition [61]. Seizures involving clinical symptoms include motor and nonmotor disorders [6,11]. Motor seizures may be automatisms, clonic, epileptic spasms, myoclonic, sequential or tonic, and nonmotor seizures may be autonomic or behavior arrest seizures [6]. According to the ILAE, the nature of seizures can be determined based on the onset (focal or generalized) [60]. However, it was found that neonatal seizures have only a focal onset, therefore, the division into focal and generalized seizures (used in the classification of seizures in the entire population) is not necessary [11,62]. There is no division into seizures with preserved consciousness and seizures with loss of consciousness due to the difficulties in reliable assessment of consciousness in neonates [11]. The following step is related to the differentiation between motor and nonmotor seizures. Finally, their type is determined [11,60]. Neonatal seizures may have different clinical manifestations. However, one symptom is usually predominant. In the group of neonates, the dominant clinical symptom is more significant as this is more likely to have clinical implications for the etiology of seizures than determination of the seizure onset zone [11]. This approach is completely different to that of other age groups [60]. It is sometimes difficult to correctly diagnose the dominant symptom, especially when seizures last longer and the sequence of symptoms can be observed, often with changing lateralization [11]. In this situation, sequential seizures are observed and are often associated with an EEG change during the seizure [11,60]. 

The neonatal period is a unique period in many respects. As a result, not all guidelines for seizure classification can be applied. Apart from the previously mentioned inability to assess consciousness in neonates, several types of seizures cannot be diagnosed due to the difficulties in communication with neonates (lack of verbal communication and significant nonverbal limitation) [11]. These types include sensory, cognitive and emotional seizures [11]. Similarly, somatosensory or visual auras cannot be determined in neonates. Due to low muscle tone, the occurrence of atonic seizures cannot be assessed clinically without invasive methods [11]. The classification of neonatal seizures including nonmotor and motor seizures is given in Table 5.

The aim of the unification of the classification system and terminology when describing individual seizures is to improve communication related to health care professionals, parents and medical care assistants. Classification also allows grouping of patients for therapies. Currently, some regulatory agencies approve drugs or medical devices indicated for specific types of seizures [60].

### 3.5. Diagnosis

Neonatal seizures are an emergency and therefore, diagnostic and therapeutic procedures should be performed simultaneously. In the diagnosis of acute symptomatic neonatal seizures, it is necessary to initially exclude reversible systemic disorders (such as electrolyte disturbances or hypoglycemia). However, if such disorders are confirmed, neuroactive anticonvulsants do not have to be administered and there is no need to perform other tests that may often be invasive. If possible, continuous video electroencephalographic (cEEG) monitoring, which is the gold standard in the diagnosis of neonatal seizures, should be immediately used [12,13]. This seems to be an ideal solution as a large proportion of seizures may be clinically silent while changes in EEG are observed. As already mentioned, it is mostly related to neonates with encephalopathy and neonates in critical condition [11]. Neonatal seizures are manifested in the EEG by a distinct bioelectric pattern that evolves in frequency, morphology or location, with a voltage of >2 μV and a duration of >10 seconds [1]. It is considered that the EEG recording requires an interval of at least 10 seconds to separate two distinct seizures [1]. Various electroclinical phenotypes may suggest specific syndromes [1]. In 2011, the American Clinical Neurophysiology Society (ACNS) developed specific guidelines related to the use of neonatal monitoring with conventional EEG [63]. ACNS distinguishes high-risk neonates among the group of neonates and thus recommends monitoring with video-EEG. Among the high-risk group of neonates, there are those with suspected or demonstrated acute brain injury and patients clinically suspected of neonatal seizures or epilepsy syndromes [63]. In addition, the high-risk group requiring EEG monitoring includes neonates after cardiopulmonary resuscitation, CNS infection and meningoencephalitis. It also includes neonates with suspected or confirmed inborn errors of metabolism, CNS trauma (maternal trauma, traumatic delivery, prolonged second stage of labor), suspected or confirmed perinatal stroke or sinovenous thrombosis [63]. Preterm neonates with additional risk factors and neonates with a genetic disease involving the CNS are also included in this group [63]. All of these high-risk neonates should be monitored with 24-hour video-EEG [31,63]. In these patients, most changes occur mainly on the first day of monitoring [61,64]. In the case of the etiology of seizures associated with HIE, monitoring neonates during therapeutic hypothermia and rewarming is indicated, despite the fact that seizures are relatively rare at that time [26,65]. Newborns with clinical symptoms should be monitored until characteristic EEG changes occur. However, EEG monitoring may be discontinued if there is no correlation between symptoms and EEG, or if symptoms resolve spontaneously [31,63]. When seizures are treated, cEEG monitoring should be continued up to 24 hours after the resolution of the acute phase [63]. This is due to the fact that the use of anticonvulsants may cause electroclinical dissociation, i.e., a condition in which clinical symptoms cease to manifest, but characteristic changes in EEG can still be observed [6,17,31,66]. This suggests that symptomatic treatment is effective, but the cause of seizures has not been eliminated yet. In the case of limited availability of video-EEG and the specialists who interpret the recording, amplitude-related-EEG (aEEG), which combines single or double EEG signals with signal processing to generate a simplified image on the monitor, can be used [57,59,67]. This monitoring tool, which has lower sensitivity than conventional EEG, provides useful information mainly on the evolution of background patterns, while its ability to detect individual seizures is low [68]. This is due to the fact that a large proportion of neonatal seizures are short and focal and are also characterized by a small amplitude. Therefore, they may not be detected by aEEG [1,69]. Amplitude-related EEG is a useful tool characterized by easy application, availability of results in real time or the possibility of observing the aEEG record, which lasts several hours, on a single screen. However, aEEG cannot be considered equivalent to cEEG monitoring [59,68]. Suspicion of a seizure in the aEEG recording requires the exclusion of artifacts. Additionally, it must be confirmed by conventional EEG [1]. To conclude, aEEG is very useful in the diagnostic process in neonatal intensive care units and in situations where EEG is unavailable or impossible to be performed, e.g., due to the lack of qualified personnel [1]. Of note, automated seizure detection algorithms are included in a number of EEG and aEEG packages, and their utility for seizure detection in neonates is variable. Therefore, they can be used only as an adjuvant tool [59,70]. 

Additionally, apart from electroencephalographic studies, neuroimaging (cranial ultrasound and magnetic resonance imaging) is also used. Cranial ultrasound is the method of choice for neuroimaging because it has many benefits. The advantages include easy availability of the equipment, the possibility of bedside examination and its use among neonates in any clinical condition [1]. Head ultrasound can visualize abnormalities in brain morphology or intracranial bleeding. However, magnetic resonance imaging is becoming more commonly used since it has been recognized as the optimal method of imaging neonatal seizures [1,71]. The difficulties associated with MRI include low availability of this examination. Additionally, clinically unstable neonates cannot be evaluated by MRI [1]. Therefore, if possible, these methods should be combined to provide better results. MRI in conjunction with cranial ultrasound can significantly facilitate the diagnosis of the etiology of neonatal seizures [1,24]. 

Next to monitoring neonates with EEG and neuroimaging studies, the complete clinical examination should also be performed. It should include the physical examination, an interview with parents (if possible) about the neonate, family history, parental diseases, information about pregnancies and deliveries, which is of crucial importance [17]. Particular attention should be paid to the presence of risk factors for HIE, stroke, intracranial bleeding or infection during pregnancy [17]. Also, information on medications taken by the mother in pregnancy and medications discontinued during this period can be of benefit. Further treatment depends on the suspected cause of seizures. If an infection of the CNS is suspected, blood and urine culture should be performed, and if the neonate is clinically stable, lumbar puncture and cerebrospinal fluid assessment may be useful. When laboratory tests and neuroimaging studies do not reveal the underlying cause of seizures, specialist tests should be considered to exclude congenital metabolic and genetic defects [17].

The clinical diagnosis of neonatal seizures and the underlying causes is a challenge for clinicians. Of note, both underdiagnosis (associated with the lack of further search for causes and delayed treatment initiation) as well as overdiagnosis (in which neonates may be administered drugs that can be potentially harmful) may be related to the deterioration of health and complications in neonates. Therefore, multidisciplinary vigilance, individual approach to each neonate and treatment compliance that limit false positive or false negative diagnosis are of crucial importance. 

### 3.6. Management

#### 3.6.1. Acute Intervention

Acute neonatal seizures are an emergency and should be treated promptly. Treatment should be initiated immediately after the diagnosis of EEG seizures [31]. Depending on the clinical context, basic therapeutic procedures (e.g., hydration, correction of electrolyte or biochemical disturbances or antibiotic therapy) should be considered before seizures are confirmed by EEG [17]. On the other hand, in high-risk neonates (e.g., with HIE or ICH) treatment should be started immediately after suspected seizures, without waiting for EEG results [17,31]. After the diagnosis of changes in EEG which are characteristic of seizures, treatment should be started with a loading dose of an anticonvulsant drug (intravenous bolus). Seizures should be immediately managed even when symptoms resolved but changes in electroencephalography are still found [31]. 

**Figure 2:**
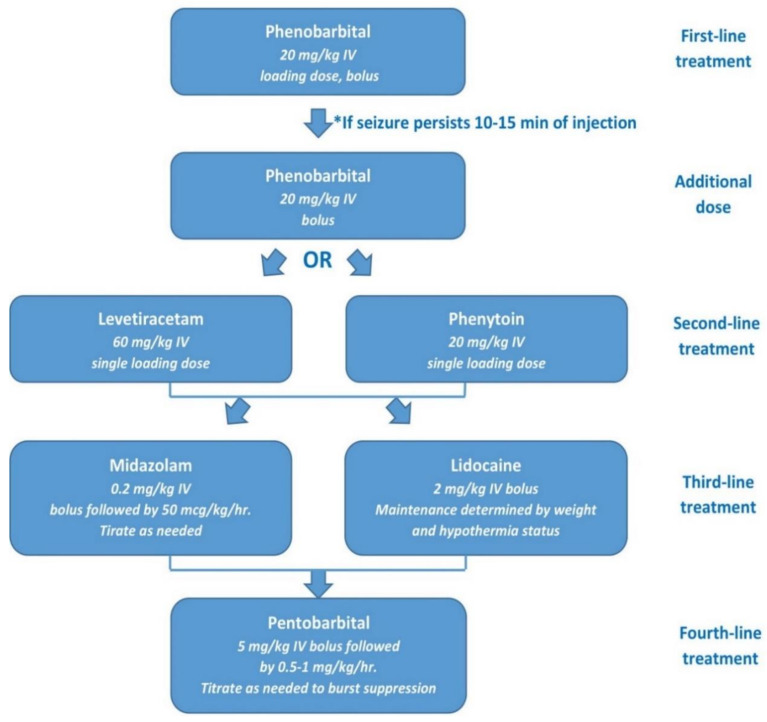
Sample algorithm for the pharmacotherapy of neonatal seizures [31].

Pharmacotherapy for neonatal seizures is empirical and varies greatly. Among many anticonvulsants, phenobarbital is the first-line drug, although its efficacy is only about 50% [2,31,72,73,74]. A single loading dose of phenobarbital is 20 mg/kg [2,59,74]. The mechanism of its action is to enhance GABA-mediated inhibition [73,75]. Phenobarbital may impair neurological development and increase neuronal apoptosis [73]. Excessive sedation, cognitive impairment or depressed mood are the most prevalent adverse effects [73,76]. Phenytoin is the second-line drug with similar efficacy to phenobarbital. It reduces neurotransmission in the brain by blocking the voltage-gated sodium channel [75]. However, it should not be used too long due to its less predictable absorption and pharmacokinetic profiles [17]. Moreover, it is related to the risk of cardiac problems and hypotension [17]. If seizures persist, a second bolus dose of the previously used drug or a loading dose of another medication is administered [59]. Levetiracetam is another drug commonly used to control neonatal seizures [17,77,78]. The loading dose is estimated at 40 mg/kg, and the maintenance dose (10 mg/kg) is administered every 8 hours [17,78,79,80]. If seizure control cannot be achieved despite the use of the abovementioned drugs, or in the case of neonatal status epilepticus, infusions of lidocaine or midazolam should be considered [17,59]. Of note, lidocaine is contraindicated in neonates who were previously treated with phenytoin or fosphenytoin. It is also contraindicated in neonates with heart defects due to its proarrhythmic effect [17,59]. Lidocaine infusion should only be continued for <30 hours [17,81]. No consensus has been reached on the duration of treatment with anticonvulsants. Some studies found that treatment with anticonvulsants can be safely discontinued after resolution of seizures in EEG recording [59,82]. However, in clinical practice, drugs are used at the lowest effective doses until the first follow-up visit [59,83]. Figure 2 shows a simplified sequence of medication use process used in the treatment of neonatal seizures.

#### 3.6.2. Treatment of Neontal Epilepsy

When the acute symptomatic cause of seizures cannot be identified and a neonate presents with symptoms of tonic seizures, neonatal epilepsy should be suspected [17]. Initially, treatment is similar to that of acute symptomatic seizures, but after the diagnosis is confirmed, more targeted treatment is used [31]. Unlike neonates with acute symptomatic seizures, neonates with epilepsy require continued pharmacotherapy after discharge home. Along with the development of molecular diagnostic methods, highly specialized targeted treatment of neonatal epilepsy has been easier to use. Neonates with structural epilepsy respond well to treatment with topiramate or oxcarbazepine [2,31,84,85,86], and those with metabolic epilepsy may require specific metabolic treatment or therapeutic administration of vitamins [31]. It has been suggested that neonates with *KCNQ2*, *KCNQ3* or *SCN2a* epilepsy respond well to treatment with low-dose sodium channel blockers, such as carbamazepine or oxcarbazepine [17,31,85,87]. Treatment should be considered before the diagnosis is confirmed in genetic tests, which is associated with better outcomes and a shorter hospitalization period [17,85]. 

### 3.7. Outcomes

The development of neonatal intensive care and the increasing availability of continuous video-EEG have significantly improved the treatment outcomes of neonates with seizures. However, this condition can still lead to disability or death. The occurrence of seizures in the neonatal period contributes to the pathological intensification of synaptic connections in the hippocampus [31]. This occurs in the post-ictal period and disrupts normal physiological synaptogenesis, which may later contribute to cognitive deficits [16,31,88]. Many risk factors for poor outcome have been identified after neonatal seizures. These include preterm birth, low birth weight, low Apgar scores, severe HIE, high-grade intraventricular hemorrhage, persistently abnormal EEG background activity, onset of seizures <24 hours or >72 hours after birth, status epilepticus, CNS infection and brain damage (detected by MRI) [2,6,89,90,91]. Depending on whether a neonate is premature or full-term, the median length of required hospitalization is different (46 and 13 days, respectively; *p* <0.0005) [2]. Interestingly, neonates who have only subclinical seizures are characterized by worse outcomes and higher mortality rates compared to neonates who have clinical manifestations with or without electrographic correlate (*p* < 0.002) [2]. Also, neonates with seizures resistant to a loading dose of medication are twice as likely to die compared to those whose seizures can be controlled with the initial loading dose of medication (*p* = 0.009) [2].

The most common neurological sequelae of neonatal seizures include developmental delay (30–50%) [6,7], epilepsy (20–35%) [6,7,8] and cerebral palsy (15–30%) [6,7,8,9]. Mortality correlates with the etiology of seizures (7–25% in neonates with seizures) [2,6,7,89,92] and is much higher in premature neonates (30–33%) [1,2,6,93,94]. Among the most common causes of seizures, the highest mortality was found in neonates with HIE (26%), ICH (13%) and ischemic stroke (4%) (*p* < 0.005) [2].

## 4. Conclusions

Although neonatal seizures are a relatively rare neurological disorder, they can be associated with severe sequelae, often affecting the patient’s normal life. Therefore, the vigilance of physicians who provide care for neonates in the first days of their lives and a rapid diagnostic process are of paramount importance. Technological progress and wider availability of video-EEG, aEEG and MRI are also crucial. To effectively treat seizures, the etiology should be determined with the greatest possible accuracy. Therefore, sometimes it is necessary to perform highly specialized and expensive metabolic or genetic tests. The key to therapeutic success seems to be the immediate management of the cause and the introduction of appropriate treatment, which will reduce adverse long-term effects and improve the results. Therefore, in certain clinical situations, treatment should be carried out simultaneously with the initial diagnosis. The reports on the approach to the diagnosis or treatment of seizures and the results obtained with the use of various therapeutic methods allow for the exchange of experience among clinicians and constitute a broad base of practical knowledge. 

## Figures and Tables

**Table 1:** Etiology and onset of neonatal seizures.

Etiology	Studies Conducted between 2013 and 2015 % Sample = 426 Neonates [2]	Studies Conducted between 2009 and 2013 % Sample = 378 Neonates [24]	Studies Conducted between 2002 and 2009 % Sample = 221 Neonates [34]	Time of Seizure Onset [31]
HIE	38	46	57.5	First 24 hours
Stroke	18	10.6	7.7	First week
ICH	12	12.2	9.0	First week
Metabolic or electrolyte disturbances	4	4.7	10.9	First few days
Infections	4	7.1	6.3	Days to weeks
Congenital CNS malformations	4	2.9	3.2	Variable
Inborn errors of metabolism	3	4.2	2.3	From day 2
Genetic epilepsy syndromes	9	2.1	2.3	Perinatal period
Unknown	9	6.3	0.5	Variable

**Table 2:** Metabolic and genetic biomarkers [1].

Disease	Urine	Plasma	CSF	Gene
PDE	Increase in AASA and PA	Increase in PA	Increase in AASA, P6C and PADecrease in PLP Sec NT abnormalities	*ALDH7A1*
PNPO	(Vanillactate)	Increase in pyridoxamine	Decrease in PLP sec NT abnormalities	*PNPO*
MOCOD, ISOD	Sulfocysteine Increase in AASA and P6C	Decrease in uric acid	Increase in AASA, P6C and PA Decrease in PLP	*MOCS1, MOCS2, GPHN*
NKH		Amino acids (glycine)	Amino acids (glycine) CSF/plasma > 0.004	4-enzyme cleavage system

Abbreviations: CSF—cerebrospinal fluid; PDE—pyridoxine-dependent epilepsy; PNPO—pyridoxal 5′-phosphate-dependent epilepsy; MOCOD—molybdenum cofactor deficiency; ISOD—isolated sulfite oxidase deficiency; NKH—nonketotic hyperglycinemia; AASA—alpha-aminoadipic semialdehyde; PA—propionic acid; P6C—piperideine 6-carboxylic acid; PLP—pyridoxal 5-phosphate; sec NT—secondary neurotransmitter.

**Table 3:** Genetic syndromes associated with neonatal seizures [37].

Type of Condition	Syndrome	Gene
Chromosomal	Down syndrome	*Trisomy 21*
	Patau syndrome	*Trisomy 13*
	Edwards syndrome	*Trisomy 18*
	22q11.2 deletion syndrome	Deletion of 22q11.2
	Wolf–Hirschhorn syndrome	Deletion of 4p16.3
Neurocutaneous	Tuberous sclerosis	*TSC1, TSC2*
	Sturge–Weber	*GNAQ*
	Incontinentia pigmenti	*IBKKG*
	Hypomelanosis of Ito	Mosaicism for aneuploidy or other chromosomal anomalies
Other	COL4A1-related	*COL4A1*
	Pitt-Hopkins	*TCF4*
	Coffin–Siris	*ARID1A, ARID1B, SMARCA4, SMARCB1, SMARCE1, SOX11*
	Aicardi–Goutieres	*ADAR, RNASEH2A, RNASEH2B, RNASEH2C, SAMHD1, TREX1, IFIH1*

**Table 4:** Types of single gene mutations in neonatal epilepsy [31,37].

Type	Time of Seizure Onset	Genetic Variants	Prognosis
Self-limited neonatal seizures	Between 4th and 6th days of life	Most unknown, *KCNQ2*	Good
Self-limited familial neonatal epilepsy	Days 2–3	Autosomal dominant in *KCNQ2, KCNQ3, SCN2A*	Good
Early infantile epileptic encephalopathy	First two weeks	Structural brain malformations, gene variants in *ARX, CDKL5, SLC25A22, STXBP1, KCNQ2, SPTAN1, SCN2A*, metabolic disorders	Frequent early-life mortality, developmental disabilities
Early myoclonic encephalopathy	Hours to months	*STXBP1, TBC1D24, GABRA1*, metabolic disorders	Frequent early-life mortality, developmental disabilities
Epilepsy of infancy with migrating focal seizures	Days to months	*KCNT1, SCN2A, SCN1A, SLC25A22, PLCB1, TBC1D24, QARS*	Poor, developmental disabilities

**Table 5:** Types of neonatal seizures including motor and nonmotor seizures [11].

Motor Seizures		Nonmotor Seizures
Seizure Type	Modifiers	Seizure Type
Automatism	Unilateral Bilateral asymmetric Bilateral symmetric	Autonomic
Clonic seizures	Focal Multifocal Bilateral	Behavioral arrest
Tonic seizures	Focal Bilateral asymmetric Bilateral symmetric	
Myoclonic seizures	Focal Multifocal Bilateral asymmetric Bilateral symmetric	
Sequential seizure type	Depending on components	
Epileptic spasm	Unilateral Bilateral asymmetric Bilateral symmetric	

## Data Availability

Not applicable.

## References

[1] Ramantani G., Schmitt B., Plecko B., Pressler R.M., Wohlrab G., Klebermass-Schrehof K., Hagmann C., Pisani F., Boylan G.B. (2019). Neonatal Seizures-Are We there Yet?. Neuropediatrics.

[2] Glass H.C., Shellhaas R.A., Wusthoff C.J., Chang T., Abend N.S., Chu C.J., Cilio M.R., Glidden D.V., Bonifacio S.L., Massey S. (2016). Contemporary Profile of Seizures in Neonates: A Prospective Cohort Study. J. Pediatr..

[3] Spagnoli C., Falsaperla R., Deolmi M., Corsello G., Pisani F. (2018). Symptomatic seizures in preterm newborns: A review on clinical features and prognosis. Ital. J. Pediatr..

[4] Glass H.C., Shellhaas R.A., Tsuchida T.N., Chang T., Wusthoff C.J., Chu C.J., Cilio M.R., Bonifacio S.L., Massey S.L., Abend N.S. (2017). Seizures in Preterm Neonates: A Multicenter Observational Cohort Study. Pediatr. Neurol..

[5] Padiyar S., Nusairat L., Kadri A., Abu-Shaweesh J., Aly H. (2020). Neonatal seizures in the U.S. National Inpatient Population: Prevalence and outcomes. Pediatr. Neonatol..

[6] Pellegrin S., Munoz F.M., Padula M., Heath P.T., Meller L., Top K., Wilmshurst J., Wiznitzer M., Das M.K., Hahn C.D. (2019). Brighton Collaboration Neonatal Seizures Working Group. Neonatal seizures: Case definition & guidelines for data collection, analysis, and presentation of immunization safety data. Vaccine.

[7] Pisani F., Piccolo B., Cantalupo G., Copioli C., Fusco C., Pelosi A., Tassinari C.A., Seri S. (2012). Neonatal seizures and postneonatal epilepsy: A 7-y follow-up study. Pediatr. Res..

[8] Yıldız E.P., Tatlı B., Ekici B., Eraslan E., Aydınlı N., Calışkan M., Ozmen M. (2012). Evaluation of etiologic and prognostic factors in neonatal convulsions. Pediatr. Neurol..

[9] Anand V., Nair P.M. (2014). Neonatal seizures: Predictors of adverse outcome. J. Pediatr. Neurosci..

[10] Abend N.S., Wusthoff C.J., Goldberg E.M., Dlugos D.J. (2013). Electrographic seizures and status epilepticus in critically ill children and neonates with encephalopathy. Lancet Neurol..

[11] Pressler R.M., Cilio M.R., Mizrahi E.M., Moshé S.L., Nunes M.L., Plouin P., Vanhatalo S., Yozawitz E., Zuberi S.M. (2018). The ILAE classification of seizures & the epilepsies: Modification for seizures in the neonate. Proposal from the ILAE task force on neonatal seizures. Epilepsia.

[12] Shellhaas R.A. (2015). Continuous long-term electroencephalography: The gold standard for neonatal seizure diagnosis. Semin. Fetal Neonatal Med..

[13] Plouin P., Kaminska A. (2013). Neonatal seizures. Handb. Clin. Neurol..

[14] Puskarjov M., Kahle K.T., Ruusuvuori E., Kaila K. (2014). Pharmacotherapeutic targeting of cation-chloride cotransporters in neonatal seizures. Epilepsia.

[15] Nardou R., Ferrari D.C., Ben-Ari Y. (2013). Mechanisms and effects of seizures in the immature brain. Semin. Fetal Neonatal Med..

[16] Carrasco M., Stafstrom C.E. (2018). How Early Can a Seizure Happen? Pathophysiological Considerations of Extremely Premature Infant Brain Development. Dev. Neurosci..

[17] Shellhaas R.A. (2019). Seizure classification, etiology, and management. Handb. Clin. Neurol..

[18] Kang S., Kadam S. (2014). Pre-Clinical Models of Acquired Neonatal Seizures: Differential Effects of Injury on Function of Chloride Co-Transporters. Austin J. Cereb. Dis. Stroke.

[19] Miller S.M., Goasdoue K., Björkman S.T. (2017). Neonatal seizures and disruption to neurotransmitter systems. Neural Regen Res..

[20] Vasudevan C., Levene M. (2013). Epidemiology and aetiology of neonatal seizures. Semin Fetal Neonatal Med..

[21] Glass H.C. (2014). Neonatal seizures: Advances in mechanisms and management. Clin Perinatol..

[22] Shah D.K., Zempel J., Barton T., Lukas K., Inder T.E. (2010). Electrographic seizures in preterm infants during the first week of life are associated with cerebral injury. Pediatr. Res..

[23] Pisani F., Facini C., Pelosi A., Mazzotta S., Spagnoli C., Pavlidis E. (2016). Neonatal seizures in preterm newborns: A predictive model for outcome. Eur. J. Paediatr. Neurol..

[24] Weeke L.C., Groenendaal F., Toet M.C., Benders M.J., Nievelstein R.A., van Rooij L.G., de Vries L.S. (2015). The aetiology of neonatal seizures and the diagnostic contribution of neonatal cerebral magnetic resonance imaging. Dev. Med. Child Neurol..

[25] Jacobs S.E., Berg M., Hunt R., Tarnow-Mordi W.O., Inder T.E., Davis P.G. (2013). Cooling for newborns with hypoxic ischaemic encephalopathy. Cochrane Database Syst. Rev..

[26] Glass H.C., Wusthoff C.J., Shellhaas R.A., Tsuchida T.N., Bonifacio S.L., Cordeiro M., Sullivan J., Abend N.S., Chang T. (2014). Risk factors for EEG seizures in neonates treated with hypothermia: A multicenter cohort study. Neurology.

[27] Boylan G.B., Kharoshankaya L., Wusthoff C.J. (2015). Seizures and hypothermia: Importance of electroencephalographic monitoring and considerations for treatment. Semin. Fetal Neonatal Med..

[28] Kirton A., Armstrong-Wells J., Chang T., Deveber G., Rivkin M.J., Hernandez M., Carpenter J., Yager J.Y., Lynch J.K., Ferriero D.M. (2011). International Pediatric Stroke Study Investigators. Symptomatic neonatal arterial ischemic stroke: The International Pediatric Stroke Study. Pediatrics.

[29] Martinez-Biarge M., Cheong J.L., Diez-Sebastian J., Mercuri E., Dubowitz L.M., Cowan F.M. (2016). Risk Factors for Neonatal Arterial Ischemic Stroke: The Importance of the Intrapartum Period. J. Pediatr..

[30] Darmency-Stamboul V., Chantegret C., Ferdynus C., Mejean N., Durand C., Sagot P., Giroud M., Bejot Y., Gouyon J.B. (2012). Antenatal factors associated with perinatal arterial ischemic stroke. Stroke.

[31] Ziobro J., Shellhaas R.A. (2020). Neonatal Seizures: Diagnosis, Etiologies, and Management. Semin Neurol..

[32] Yamamoto H., Okumura A., Fukuda M. (2011). Epilepsies and epileptic syndromes starting in the neonatal period. Brain Dev..

[33] Oh A., Thurman D.J., Kim H. (2019). Independent role of neonatal seizures in subsequent neurological outcomes: A population-based study. Dev. Med. Child Neurol..

[34] Loman A.M., ter Horst H.J., Lambrechtsen F.A., Lunsing R.J. (2014). Neonatal seizures: Aetiology by means of a standardized work-up. Eur. J. Paediatr. Neurol..

[35] Axeen E.J.T., Olson H.E. (2018). Neonatal epilepsy genetics. Semin. Fetal Neonatal Med..

[36] Shellhaas R.A., Wusthoff C.J., Tsuchida T.N., Glass H.C., Chu C.J., Massey S.L., Soul J.S., Wiwattanadittakun N., Abend N.S., Cilio M.R. (2017). Neonatal Seizure Registry. Profile of neonatal epilepsies: Characteristics of a prospective US cohort. Neurology.

[37] Kaur S., Pappas K. (2020). Genetic Etiologies of Neonatal Seizures. Neoreviews.

[38] Cornet M.C., Sands T.T., Cilio M.R. (2018). Neonatal epilepsies: Clinical management. Semin. Fetal Neonatal Med..

[39] Ficicioglu C., Bearden D. (2011). Isolated neonatal seizures: When to suspect inborn errors of metabolism. Pediatr. Neurol..

[40] Dulac O., Plecko B., Gataullina S., Wolf N.I. (2014). Occasional seizures, epilepsy, and inborn errors of metabolism. Lancet Neurol..

[41] Scheffer I.E. (2014). Epilepsy genetics revolutionizes clinical practice. Neuropediatrics.

[42] Mills P.B., Footitt E.J., Mills K.A., Tuschl K., Aylett S., Varadkar S., Hemingway C., Marlow N., Rennie J., Baxter P. (2010). Genotypic and phenotypic spectrum of pyridoxine-dependent epilepsy (ALDH7A1 deficiency). Brain.

[43] Pearl P.L. (2016). Amenable Treatable Severe Pediatric Epilepsies. Semin. Pediatr. Neurol..

[44] Campistol J. (2016). Epilepsy in Inborn Errors of Metabolism with Therapeutic Options. Semin. Pediatr. Neurol..

[45] Mastrangelo M. (2018). Actual Insights into Treatable Inborn Errors of Metabolism Causing Epilepsy. J. Pediatr. Neurosci..

[46] Sharma S., Prasad A.N. (2017). Inborn Errors of Metabolism and Epilepsy: Current Understanding, Diagnosis, and Treatment Approaches. Int. J. Mol. Sci..

[47] Stence N.V., Coughlin C.R., Fenton L.Z., Thomas J.A. (2013). Distinctive pattern of restricted diffusion in a neonate with molybdenum cofactor deficiency. Pediatr. Radiol..

[48] Hannah-Shmouni F., MacNeil L., Potter M., Jobling R., Yoon G., Laughlin S., Blaser S., Inbar-Feigenberg M. (2018). Severe cystic degeneration and intractable seizures in a newborn with molybdenum cofactor deficiency type B. Mol. Genet. Metab. Rep..

[49] Sands T.T., McDonough T.L. (2016). Recent Advances in Neonatal Seizures. Curr. Neurol. Neurosci. Rep..

[50] Liu J., Tong L., Song S., Niu Y., Li J., Wu X., Zhang J., Zai C.C., Luo F., Wu J. (2018). Novel and de novo mutations in pediatric refractory epilepsy. Mol. Brain..

[51] Maljevic S., Vejzovic S., Bernhard M.K., Bertsche A., Weise S., Döcker M., Lerche H., Lemke J.R., Merkenschlager A., Syrbe S. (2016). Novel *KCNQ3* Mutation in a Large Family with Benign Familial Neonatal Epilepsy: A Rare Cause of Neonatal Seizures. Mol. Syndromol..

[52] Saadeldin I.Y., Milhem R.M., Al-Gazali L., Ali B.R. (2013). Novel *KCNQ2* mutation in a large Emirati family with benign familial neonatal seizures. Pediatric Neurol..

[53] Pearl P.L. (2018). Epilepsy Syndromes in Childhood. Continuum (Minneap Minn). Child Neurol..

[54] Hart A.R., Pilling E.L., Alix J.J. (2015). Neonatal seizures-part 2: Aetiology of acute symptomatic seizures, treatments and the neonatal epilepsy syndromes. Arch. Dis. Child Educ. Pract. Ed..

[55] Beal J.C., Cherian K., Moshe S.L. (2012). Early-onset epileptic encephalopathies: Ohtahara syndrome and early myoclonic encephalopathy. Pediatr. Neurol..

[56] McTague A., Appleton R., Avula S., Cross J.H., King M.D., Jacques T.S., Bhate S., Cronin A., Curran A., Desurkar A. (2013). Migrating partial seizures of infancy: Expansion of the electroclinical, radiological and pathological disease spectrum. Brain.

[57] Ma X., Yang F., Hua Z. (2019). Genetic diagnosis of neonatal-onset seizures. Genes Dis..

[58] Hallberg B., Blennow M. (2013). Investigations for neonatal seizures. Semin. Fetal Neonatal Med..

[59] Glass H.C., Shellhaas R.A. (2019). Acute Symptomatic Seizures in Neonates. Semin. Pediatr. Neurol..

[60] Fisher R.S., Cross J.H., French J.A., Higurashi N., Hirsch E., Jansen F.E., Lagae L., Moshé S.L., Peltola J., Roulet Perez E. (2017). Operational classification of seizure types by the International League Against Epilepsy: Position Paper of the ILAE Commission for Classification and Terminology. Epilepsia.

[61] Nash K.B., Bonifacio S.L., Glass H.C., Sullivan J.E., Barkovich A.J., Ferriero D.M., Cilio M.R. (2011). Video-EEG monitoring in newborns with hypoxic-ischemic encephalopathy treated with hypothermia. Neurology.

[62] Nagarajan L., Ghosh S., Palumbo L. (2011). Ictal electroencephalograms in neonatal seizures: Characteristics and associations. Pediatr. Neurol..

[63] Shellhaas R.A., Chang T., Tsuchida T., Scher M.S., Riviello J.J., Abend N.S., Nguyen S., Wusthoff C.J., Clancy R.R. (2011). The American Clinical Neurophysiology Society’s Guideline on Continuous Electroencephalography Monitoring in Neonates. J. Clin. Neurophysiol..

[64] Benedetti G.M., Silverstein F.S., Rau S.M., Lester S.G., Benedetti M.H., Shellhaas R.A. (2018). Sedation and Analgesia Influence Electroencephalography Monitoring in Pediatric Neurocritical Care. Pediatr. Neurol..

[65] Cornet M.C., Pasupuleti A., Fang A., Gonzalez F., Shimotake T., Ferriero D.M., Glass H.C., Cilio M.R. (2018). Predictive value of early EEG for seizures in neonates with hypoxic-ischemic encephalopathy undergoing therapeutic hypothermia. Pediatr. Res..

[66] Mathieson S.R., Livingstone V., Low E., Pressler R., Rennie J.M., Boylan G.B. (2016). Phenobarbital reduces EEG amplitude and propagation of neonatal seizures but does not alter performance of automated seizure detection. Clin. Neurophysiol..

[67] Rakshasbhuvankar A., Paul S., Nagarajan L., Ghosh S., Rao S. (2015). Amplitude-integrated EEG for detection of neonatal seizures: A systematic review. Seizure.

[68] Glass H.C., Wusthoff C.J., Shellhaas R.A. (2013). Amplitude-integrated electro-encephalography: The child neurologist’s perspective. J. Child Neurol..

[69] Shellhaas R.A., Barks A.K. (2012). Impact of amplitude-integrated electroencephalograms on clinical care for neonates with seizures. Pediatr. Neurol..

[70] Mathieson S., Rennie J., Livingstone V., Temko A., Low E., Pressler R.M., Boylan G.B. (2016). In-depth performance analysis of an EEG based neonatal seizure detection algorithm. Clin. Neurophysiol..

[71] Weeke L.C., Van Rooij L.G., Toet M.C., Groenendaal F., de Vries L.S. (2015). Neuroimaging in neonatal seizures. Epileptic Disord..

[72] World Health Organization (2011). Guidelines on Neonatal Seizures.

[73] Donovan M.D., Griffin B.T., Kharoshankaya L., Cryan J.F., Boylan G.B. (2016). Pharmacotherapy for Neonatal Seizures: Current Knowledge and Future Perspectives. Drugs.

[74] Glass H.C., Kan J., Bonifacio S.L., Ferriero D.M. (2012). Neonatal seizures: Treatment practices among term and preterm infants. Pediatr. Neurol..

[75] Bialer M., White H.S. (2010). Key factors in the discovery and development of new antiepileptic drugs. Nat. Rev. Drug Discov..

[76] Brodie M.J., Kwan P. (2012). Current position of phenobarbital in epilepsy and its future. Epilepsia.

[77] Ahmad K.A., Desai S.J., Bennett M.M., Ahmad S.F., Ng Y.T., Clark R.H., Tolia V.N. (2017). Changing antiepileptic drug use for seizures in US neonatal intensive care units from 2005 to 2014. J. Perinatol..

[78] Ramantani G., Ikonomidou C., Walter B., Rating D., Dinger J. (2011). Levetiracetam: Safety and efficacy in neonatal seizures. Eur. J. Paediatr. Neurol..

[79] Merhar S.L., Schibler K.R., Sherwin C.M., Meinzen-Derr J., Shi J., Balmakund T., Vinks A.A. (2011). Pharmacokinetics of levetiracetam in neonates with seizures. J. Pediatr..

[80] Sharpe C.M., Capparelli E.V., Mower A., Farrell M.J., Soldin S.J., Haas R.H. (2012). A seven-day study of the pharmacokinetics of intravenous levetiracetam in neonates: Marked changes in pharmacokinetics occur during the first week of life. Pediatr. Res..

[81] Weeke L.C., Toet M.C., van Rooij L.G., Groenendaal F., Boylan G.B., Pressler R.M., Hellström-Westas L., van den Broek M.P., de Vries L.S. (2016). Lidocaine response rate in aEEG-confirmed neonatal seizures: Retrospective study of 413 full-term and preterm infants. Epilepsia.

[82] Fitzgerald M.P., Kessler S.K., Abend N.S. (2017). Early discontinuation of antiseizure medications in neonates with hypoxic-ischemic encephalopathy. Epilepsia.

[83] Shellhaas R.A., Chang T., Wusthoff C.J., Soul J.S., Massey S.L., Chu C.J., Cilio M.R., Bonifacio S.L., Abend N.S., Tsuchida T.N. (2017). Treatment Duration After Acute Symptomatic Seizures in Neonates: A Multicenter Cohort Study. J. Pediatr..

[84] Van Rooij L.G., van den Broek M.P., Rademaker C.M., de Vries L.S. (2013). Clinical management of seizures in newborns: Diagnosis and treatment. Paediatr. Drugs.

[85] Sands T.T., Balestri M., Bellini G., Mulkey S.B., Danhaive O., Bakken E.H., Taglialatela M., Oldham M.S., Vigevano F., Holmes G.L. (2016). Rapid and safe response to low-dose carbamazepine in neonatal epilepsy. Epilepsia.

[86] El-Dib M., Soul J.S. (2017). The use of phenobarbital and other anti-seizure drugs in newborns. Semin. Fetal Neonatal Med..

[87] Numis A.L., Angriman M., Sullivan J.E., Lewis A.J., Striano P., Nabbout R., Cilio M.R. (2014). KCNQ2 encephalopathy: Delineation of the electroclinical phenotype and treatment response. Neurology.

[88] O’Leary H., Bernard P.B., Castano A.M., Benke T.A. (2016). Enhanced long term potentiation and decreased AMPA receptor desensitization in the acute period following a single kainate induced early life seizure. Neurobiol. Dis..

[89] Glass H.C., Grinspan Z.M., Shellhaas R.A. (2018). Outcomes after acute symptomatic seizures in neonates. Semin. Fetal Neonatal Med..

[90] Kharoshankaya L., Stevenson N.J., Livingstone V., Murray D.M., Murphy B.P., Ahearne C.E., Boylan G.B. (2016). Seizure burden and neurodevelopmental outcome in neonates with hypoxic-ischemic encephalopathy. Dev. Med. Child Neurol..

[91] Pinchefsky E.F., Hahn C.D. (2017). Outcomes following electrographic seizures and electrographic status epilepticus in the pediatric and neonatal ICUs. Curr. Opin. Neurol..

[92] Mwaniki M., Mathenge A., Gwer S., Mturi N., Bauni E., Newton C.R., Berkley J., Idro R. (2010). Neonatal seizures in a rural Kenyan District Hospital: Aetiology, incidence and outcome of hospitalization. BMC Med..

[93] Pisani F., Copioli C., Turco E.C., Sisti L., Cossu G., Seri S. (2012). Mortality risk after neonatal seizures in very preterm newborns. J. Child Neurol..

[94] Heljic S., Uzicanin S., Catibusic F., Zubcevic S. (2016). Predictors of Mortality in Neonates with Seizures; a Prospective Cohort Study. Med. Arch..

